# On the impact of model selection on predictor identification and parameter inference

**DOI:** 10.1007/s00180-016-0690-2

**Published:** 2016-10-22

**Authors:** Ruth M. Pfeiffer, Andrew Redd, Raymond J. Carroll

**Affiliations:** 1 Division of Cancer Epidemiology and Genetics, National Cancer Institute, 9609 Medical Center Drive, Room 7E142, Bethesda, MD 20892 USA; 2 Department of Internal Medicine, University of Utah School of Medicine, Salt Lake City, UT 84132 USA; 3 Department of Statistics, Texas A&M University, College Station, TX 77843-3143 USA

**Keywords:** Biased estimates, Post-model selection inference, Finite sample inference, Shrinkage, Variable selection

## Abstract

We assessed the ability of several penalized regression methods for linear and logistic models to identify outcome-associated predictors and the impact of predictor selection on parameter inference for practical sample sizes. We studied effect estimates obtained directly from penalized methods (Algorithm 1), or by refitting selected predictors with standard regression (Algorithm 2). For linear models, penalized linear regression, elastic net, smoothly clipped absolute deviation (SCAD), least angle regression and LASSO had a low false negative (FN) predictor selection rates but false positive (FP) rates above 20 % for all sample and effect sizes. Partial least squares regression had few FPs but many FNs. Only relaxo had low FP and FN rates. For logistic models, LASSO and penalized logistic regression had many FPs and few FNs for all sample and effect sizes. SCAD and adaptive logistic regression had low or moderate FP rates but many FNs. 95 % confidence interval coverage of predictors with null effects was approximately 100 % for Algorithm 1 for all methods, and 95 % for Algorithm 2 for large sample and effect sizes. Coverage was low only for penalized partial least squares (linear regression). For outcome-associated predictors, coverage was close to 95 % for Algorithm 2 for large sample and effect sizes for all methods except penalized partial least squares and penalized logistic regression. Coverage was sub-nominal for Algorithm 1. In conclusion, many methods performed comparably, and while Algorithm 2 is preferred to Algorithm 1 for estimation, it yields valid inference only for large effect and sample sizes.

**Electronic supplementary material:**

The online version of this article (doi:10.1007/s00180-016-0690-2) contains supplementary material, which is available to authorized users.

## Introduction

Many regression procedures have been proposed in the recent literature that use penalties on regression coefficients in order to achieve sparseness or shrink them toward zero. These methods are popular for the analysis of datasets with large numbers of predictors, as they allow efficient selection of regression variables. While in many applications the primary interest is in identifying outcome associated covariates, it is nonetheless sometimes also desirable to gain scientific insights into the data generating process, and draw statistical inference on the parameters associated with the selected variables. Biases in estimates as well as in standard errors and confidence intervals become important if investigators focus on the magnitude of the observed effects.

Selection of predictor variables is a special case of model selection, which can be stated as follows. Let $\mathcal {M}$ denote the space of all candidate models that could be used to describe the data *D*. For our purposes $\mathcal {M}$ is characterized in terms of distribution functions that depend on parameters ${\varvec{\beta }}$ and may or may not contain the true model that gave rise to the data. The model selection problem is to choose a model $\hat{M}(D)$ in $\mathcal {M}$ such that $\hat{M}$ is a “good” model in terms of parameter estimation or prediction. If the focus is on inference regarding the parameters, then the quantity of interest is $\hat{{\varvec{\beta }}}(\hat{M})$. Model selection is a source of variability that is often ignored in standard statistical approaches. However, several authors, e.g. Sen (1979), Pötscher (1991) and Leeb (2005), have shown that the asymptotic distribution of the post-model selection estimates $n^{1/2} (\hat{{\varvec{\beta }}}-{\varvec{\beta }})$, where *n* denotes the sample size, is typically non-normal, and depends on the unknown ${\varvec{\beta }}$ in complex fashions.

Some analytical results are available for penalized maximum likelihood estimators obtained from LASSO (Tibshirani 1996), SCAD (smoothly clipped absolute deviation; Fan and Li 2001) and hard thresholding for linear regression models, see e.g. Knight and Fu (2000), Leeb and Pötscher (2009) and Pötscher and Schneider (2009). These estimators have highly non-normal finite sample distributions and under conservative model selection their large sample distribution can be far from normal. Even under consistent model selection (pointwise) asymptotic analysis gives highly misleading results. In addition, the large sample properties depend on the choice of tuning parameters. Therefore the naively estimated standard error for those estimates will be biased and confidence intervals based on standard asymptotic theory for these methods may not have proper coverage, not even asymptotically.

No comprehensive comparisons of penalized approaches with respect to their finite sample properties have been performed to date, and little work has been done for non-linear models. We thus studied the properties of estimates obtained from popular penalized likelihood approaches applied to linear and logistic regression models using simulated data, focusing on realistic effect and sample sizes to make conclusions applicable to practical settings (Sect. 2.2). We first assess the methods’ ability to identify truly outcome-associated predictors. We then study properties of effect estimates obtained directly from penalized methods (Algorithm 1), or by refitting selected predictors with standard regression (Algorithm 2) described in Sect. 2.3. The results presented in Sect. 3 can help to avoid overly optimistic interpretation of parameters in future research.

## Methods

The goal of this analysis is to assess the impact of model selection on parameter estimates in linear and logistic models. We evaluated the influence of sample size, *n*, and magnitude of the regression coefficients for associations ${{\varvec{\beta }}}$ on each method’s ability to identify outcome associated predictors. We also studied properties of effect estimates obtained directly from penalized methods (Algorithm 1), or by refitting selected predictors with standard regression (Algorithm 2). A range of sample sizes, algorithms and correlation structures among predictors are utilized.

### Estimation methods and algorithms

We calculated both the LASSO (Tibshirani 1996), and least angle regression (LARS, Efron et al. 2004) estimates with the function lars in the lars library of the statistical package R (Ihaka and Gentlemen 1996). The elastic net was fit using the function enet in the elasticnet library (Zou and Hastie 2005). We used the function relaxo in the library relaxo in R to fit relaxed LASSO, a generalization of the LASSO shrinkage technique (Meinshausen 2007). Generalized linear model (GLM) estimates with L1 (LASSO) and/or L2 (ridge) penalties, or a combination are obtained using the library and function penalized (Goeman 2010).

To fit L2 penalized logistic regression models with a stepwise variable selection, we used the function plr in the package stepPlr (Park and Hastie 2008). We also used an *R* implementation of SCAD, available at http://www.stat.umn.edu/~hzou/ftpdir/code/one-step-SCAD-funs.R (accessed 05/09).

For linear and binary outcome data regression coefficients for penalized partial least squares were obtained using the function penalized.pls in the library ppls (Krämer et al. 2008).

We used fivefold cross validation to select tuning parameters for all the methods that allowed that option.

Table 1 summarizes the algorithms and software packages.

**Table 1:** Algorithms and software used in the simulation study

Algorithm	Software
LASSO	R library lars
relaxed LASSO	R library relaxo
LARS	R library lars
elastic net	R library elasticnet
SCAD	http://www.stat.umn.edu/~hzou/ftpdir/code/one-step-SCAD-funs.R (accessed 05/09)
GLM with L1 and/or L2 penalties	R library penalized
Penalized partial least squares	R library ppls
Logistic regr. w L2 penalty and stepwise selection	R library stepPlr

### Simulated data

#### Continuous outcome data

Each observation in a data set of size *n* contains the predictors $\mathbf{X} =(X_1, \ldots , X_p)'$, and the continuous outcome variable, *Y*. We assumed that only a small number of predictors $p^*<p$ are associated with *Y* and denote those by $\mathbf{X}^*=(X^*_{1}, \ldots , X^*_{p^*})'$, a $1\times p^*$ subvector of $\mathbf{X}$. For ease of exposition we let the predictors in $\mathbf{X}$ be ordered so that the first $p^*$ values of $\mathbf{X}$ correspond to $\mathbf{X}^*$. Given $\mathbf{X}^*$ and ${\varvec{\beta }}^*=(\beta ^*_{1},\ldots ,\beta ^*_{p^*})'$, a $1\times p^*$ vector with $\beta _i^* \ne 0$, the response *Y* was generated from the linear model

1 $$\begin{aligned} Y = \beta ^*_0+ \mathbf{X^*}' {\varvec{\beta }}^* + \epsilon ,~~\epsilon \sim \mathcal{N}(0,\sigma ^2_*). \end{aligned}$$

For each simulation, we then fit a linear model using all available predictors, $\mathbf{X}$, i.e. assuming

2 $$\begin{aligned} Y =\beta _0+\mathbf{X}' {\varvec{\beta }}+ \epsilon ,~~\epsilon \sim \mathcal{N}(0,\sigma ^2), \end{aligned}$$

using the methods given in Sect. 2.1 and obtained the $1\times p$ vector of parameter estimates $\hat{{\varvec{\beta }}}$ of ${\varvec{\beta }}$. In all settings we studied, $p=50$ and $p^*=10$ and the variance of the error term in Eq. (1) was $\sigma ^2_*=1$. We also assessed the robustness of the methods by generating $\epsilon$ from a t-distribution with two degrees of freedom.

We generated $\mathbf{X}$ from a multivariate normal distribution, with mean $\mathbf {0}$ and correlation matrix $\Sigma _X$. To assess the impact of various correlation structures among the predictors on the performance of the methods, we studied several choices of $\Sigma _X = (\sigma _{ij}), i,j=1,\ldots ,p$. They include the independence correlation structure, $\Sigma _X=\mathbf {I}$, where $\mathbf {I}$ denotes the $p \times p$ identity matrix, a block diagonal structure for $\Sigma _X$, where each block submatrix has dimension $5 \times 5$ and constant entries $\sigma _{ij}=0.5, i \ne j$ for $|i-j| \le 5$, and $\sigma _{ij}= 0$ otherwise, and an autoregressive (AR) correlation structure for $\Sigma _X$ with $\sigma _{ij}= 0.5^{|i-j|}$ for $|i-j| \le 10$ and $\sigma _{ij}= 0$ otherwise.

#### Binary outcome data

Binary data, labeled ”controls” ($Y=0$) and ”cases” ($Y=1$), were simulated similarly to the continuous outcomes. The probability $P(Y=1)$ was a function of the predictors $\mathbf{X}^*$, the $p^*$ dimensional subvector of $\mathbf{X}$:

3 $$\begin{aligned} \hbox {logit }\{ P(Y = 1| \mathbf{X}^*)\} = \beta _0^*+ \mathbf{X}^{*'} {\varvec{\beta }}^*, \end{aligned}$$

where logit$(x) = \exp (x) / \{ 1 + \exp (x)\}$. For each simulation we created a population of subjects by drawing *Y* from a Bernoulli distribution with success probability given by model (3) with $\beta _0^*=-1$, given $\mathbf{X}$, and then sampled a fixed number of cases and controls to obtain a case-control study, a design popular in biological applications. Again, $p=50$ and $p^*=10$.

For each simulation, we obtained estimates $\hat{{\varvec{\beta }}}$ by fitting a logistic model using all available predictors, $\mathbf{X}$, i.e. assuming

4 $$\begin{aligned} \hbox {logit }\{P(Y = 1| \mathbf{X})\} = \beta _0+ \mathbf{X}^{'} {\varvec{\beta }}\end{aligned}$$

using the methods given in Sect. 2.1.

We study multivariate normally distributed predictors $\mathbf{X}$ that have mean zero and $\Sigma _X=\mathbf {I},$ and also binary predictors $\mathbf{X}$, that is $X_{i}=0$ or $X_{i}=1$, with $P(X_{i}=1)=0.5$.

#### Parameter choices and sample sizes

For simplicity, we assume that all outcome associated ${\varvec{\beta }}^*=(\beta ^*_1,\ldots ,\beta ^*_{p*})$ coefficients in models (1) and (3) have the same magnitude, but half of them are positively and half of them are negatively associated with *Y*, i.e. the $\beta ^*_i$s differ by their sign. We chose $\beta _i^*=0.25, 0.5$ and 1.0 for both linear and logistic models. For continuous outcomes the sample sizes were $n=100$, $n=200$, and $n=500$. For the binary outcome setting we used a case-control design with equal numbers of controls ($n_0$) and cases ($n_1$) with $n_0=n_1=100$, $n_0=n_1=200$, and $n_0=n_1=500$. All simulations and analyses were implemented in R.

### Analysis 

We assessed the performance of two strategies to obtain parameter estimates and their standard errors for both linear and logistic regression models.

#### Linear regression

##### Algorithm 1

(*Adaptive approach*)

This is a one-stage approach that uses the estimates $\hat{{\varvec{\beta }}}$ obtained from the respective procedure. We denote the vector of coefficients of $\hat{{\varvec{\beta }}}$ that are either the intercept or are non-zero by $\hat{{\varvec{\beta }}}_{{ adapt}}$, and by $\hat{X}_{Si}$ the vector of predictors for the $i^{th}$ subject corresponding to the intercept and the non-zero parameter estimates. The corresponding $p_{{ adapt}} \times n$ design matrix is $\hat{\mathbf{X}}_S$. We let $\hat{\sigma }_{{ adapt}}^{2}$ be the mean squared error of the fit for $\hat{{\varvec{\beta }}}$, but with the degrees of freedom $n-p_{{ adapt}}$, where $p_{{ adapt}}$ is the dimension of $\hat{{\varvec{\beta }}}_{{ adapt}}$. The covariance matrix of $\hat{{\varvec{\beta }}}_{{ adapt}}$ is estimated as

5 $$\begin{aligned} \widehat{cov}\left( \hat{{\varvec{\beta }}}_{{ adapt}}\right) =\hat{\sigma }_{{ adapt}}^{2}\left( \hat{\mathbf{X}}_{S}\hat{\mathbf{X}}_{S}'\right) ^{-1}. \end{aligned}$$

##### Algorithm 2

(*Oracle approach*)

This is a two stage approach. First we obtain $\hat{\mathbf{X}}_{S}$ as in Algorithm 1. In stage two we regress *Y* on $\hat{\mathbf{X}}_{S}$ to get a $p_{{ adapt}} \times 1$ vector $\hat{{\varvec{\beta }}}_{{ oracle}}$ of new parameter estimates, which include an intercept. We let $\hat{\sigma }_{{ oracle}}^{2}$ be the mean squared error of the fit when $\hat{{\varvec{\beta }}}_{{ oracle}}$ is used, with $n-p_{{ adapt}}$ degrees of freedom. The estimated covariance matrix of $\hat{{\varvec{\beta }}}_{{ oracle}}$ is then

6 $$\begin{aligned} \widehat{cov}\left( \hat{{\varvec{\beta }}}_{{ oracle}}\right) =\hat{\sigma }_{{ oracle}}^{2} \left( \hat{\mathbf{X}}_{S}\hat{\mathbf{X}}_{S}'\right) ^{-1}. \end{aligned}$$

#### Logistic regression

Like for linear regression, $\hat{X}_{Si}$ is the vector of predictors for the $i^{th}$ subject corresponding to the intercept and the non-zero components of $\hat{{\varvec{\beta }}}$ and $\hat{\mathbf{X}}_{S}$ is the corresponding design matrix.

##### Algorithm 1

(*Adaptive approach*)

Again, $\hat{{\varvec{\beta }}}_{{ adapt}}$ denotes the vector of coefficients of $\hat{{\varvec{\beta }}}$ obtained from the respective procedure that are either the intercept or are non-zero. Letting $\hbox {logit }(\hat{p}_i^a) = \hat{{X}}_{Si}'\hat{{\varvec{\beta }}}_{{ adapt}},$ $\hat{\mathbf{p}}_{{ adapt}}=(\hat{p}_1^a (1-\hat{p}_1^a),\ldots ,\hat{p}_n^a(1-\hat{p}_n^a))$ and $\hat{V}_{{ adapt}}= \hat{\mathbf{p}}_{{ adapt}} \mathbf {I}$, where $\mathbf {I}$ denotes the identity matrix, we compute the covariance matrix of the estimates as

7 $$\begin{aligned} \widehat{cov}\left( \hat{{\varvec{\beta }}}_{{ adapt}}\right) =\left( \hat{\mathbf{X}}_{S} \hat{V}_{{ adapt}} \hat{\mathbf{X}}_{S}'\right) ^{-1}. \end{aligned}$$

##### Algorithm 2

(*Oracle approach*)

First we obtain $\hat{\mathbf{X}}_{S}$, and then compute $\hat{{\varvec{\beta }}}_{{ oracle}}$ by re-fitting the standard logistic regression model with $\hat{\mathbf{X}}_{S}$ instead of $\mathbf{X}$ to the outcome data. Letting $\hbox {logit }(p_i^o) = \hat{{X}}_{Si}' \hat{{\varvec{\beta }}}_{{ oracle}}$, $\hat{\mathbf{p}}_{{ oracle}}=(\hat{p}_1^o(1-\hat{p}_1^o),\ldots ,\hat{p}_n^o(1-\hat{p}_n^o))$ and $\hat{V}_{{ oracle}}= \hat{\mathbf{p}}_{{ oracle}} \mathbf {I}$, where $\mathbf {I}$ denotes the identity matrix, we compute the covariance matrix of the estimates as

8 $$\begin{aligned} \widehat{cov}\left( \hat{{\varvec{\beta }}}_{{ oracle}}\right) =\left( \hat{\mathbf{X}}_{S} \hat{V}_{{ oracle}} \hat{\mathbf{X}}_{S}'\right) ^{-1}. \end{aligned}$$

### Performance criteria

We evaluated the influence of sample size, *n*, and magnitude of the associations ${\varvec{\beta }}^*$ on each method’s ability to identify the true outcome associated predictors $\mathbf{X}^*$ and on the two algorithms described above to estimate the corresponding regression parameters ${\varvec{\beta }}^*$.

#### Performance criteria for variable selection

*False positives (FPs)* Let ${\varvec{\beta }}=(\beta _{0},\beta _{1},\dots ,\beta _{p})'=({\varvec{\beta }}^*,\mathbf {0})'$, where $\mathbf {0}$ is a $1\times (p-p^*)$ vector of zeros. A FP occurs for $\beta _{j}$ when $\beta _{j}=0$ but its regularized estimate $\hat{\beta }_j \ne 0$. The FP rate for $\beta _{j}$ is the percentage of times an FP occurs for $\beta _{j}$, and the overall FP rate is the average of the FP rates across all zero coefficients of ${\varvec{\beta }}$.

*False Negatives (FNs) * A FN occurs for $\beta _{j}$ when $\beta _{j}\ne 0$ but its regularized estimate $\hat{\beta }_j =0$. The FN rate for $\beta _{j}$ is the percentage of times a FN occurs for $\beta _{j}$, and the overall FN rate is the average of the FN rates across all non-zero coefficients of ${\varvec{\beta }}$.

#### Impact of model selection on parameter estimates, coverage computations

The following coverages of the 95 % confidence intervals (CIs) for linear and logistic models were computed. The coverage of zeros is the number of times that either the regularized estimate of a $\beta _j=0$ coefficient is zero, i.e. $\hat{\beta }_j= 0$, or the 95 % CI of $\hat{\beta }_j \ne 0$ includes zero divided by the number of $p-p^*$ of zero coefficients. The 95 % CIs are computed using the asymptotic approximation, the normal distribution, and the standard errors from either Algorithm 1 or Algorithm 2. We compute the coverage of zeros separately for the zero and non-zero coefficients of ${\varvec{\beta }}$ and report the average over all $\beta _{j}=0$ and $\beta _{j}=\beta _j^* \ne 0$ respectively.

The coverage of the true ${\varvec{\beta }}^*$ coefficients is the number of times that the 95 % CI around $\hat{\beta }_{{ adapt}}$ or $\hat{\beta }_{{ oracle}}$ includes the true value of $\beta _j^*(=\beta _j \ne 0)$ divided by the number $p^*$ of non-zero coefficients ${\varvec{\beta }}^*$. Again, we report the average over all coefficients $\beta _j^*, j=1,\ldots , p^*$.

## Results

### Results for linear regression

*LARS*

**Fig. 1:**
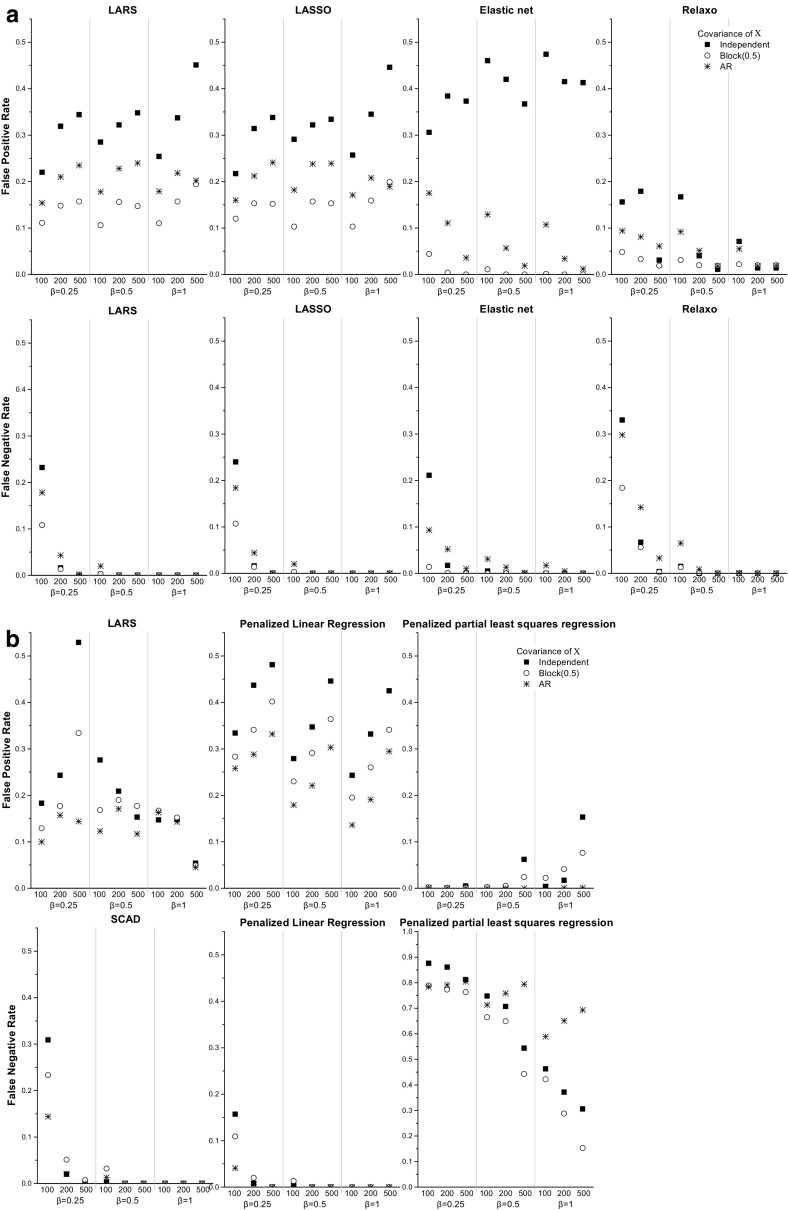
False positive (*FP*) and false negative (*FN*) rates for LARS, LASSO, elastic net and relaxo (**a**), and SCAD, penalized linear regression and penalized partial least squares regression (**b**)

**Fig. 2:**
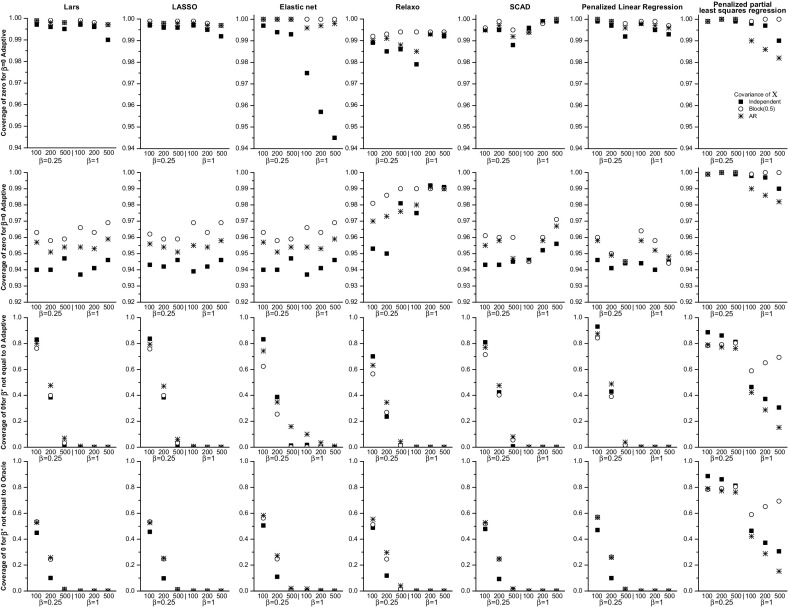
Coverage of zero for $\beta =0$ for the adaptive and oracle confidence intervals (*top two rows*) and coverage of zero for $\beta ^*\ne 0$ (*bottom two rows*)

**Fig. 3:**
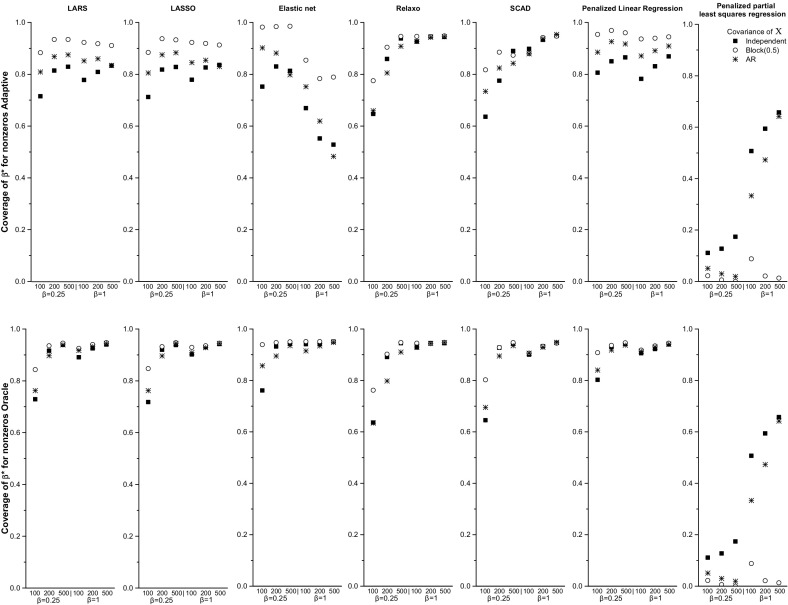
Coverage of the 95 % CIs computed based on the adaptive (Algorithm 1) or oracle (Algorithm 2) variance estimates of $\beta ^*$ for $\hat{\beta } \ne 0$

The *FP* rate ranged from 11.1 to 34.8 %, and was slightly lower for the block correlation structure than the AR or independent correlations (Fig. 1 and Supplemental Table 1). The *FN* rate was below 0.05 for sample sizes $n=200$ and $n=500$ and around 20 % for $n=100$ for all effect sizes and correlations. The coverage of zero for $\beta _j= 0$ was close to 100 % for Algorithm 1 and around 95 % for Algorithm 2. The coverage of zero for $\beta ^*\ne 0$ was 0 % for both algorithms for $n=500$ for all effect sizes (Fig. 2). The 95 % CI coverage of $\beta ^*$ for the $\hat{\beta } \ne 0$ coefficients was around 95 % for Algorithm 2 with $n=500$. It also was around 95 % for both algorithms for $n=200$ and $n=500$ for the block correlation structure, but for all other correlations Algorithm 1 had lower coverage than Algorithm 2, generally below 90 % (Fig. 3).

*LASSO*

Similar to *LARS*, the *FP* rate was slightly lower for the block correlation structure than the AR or independent correlations (Fig. 1 and Supplemental Table 2), and it ranged from 10.3 to 44.6 %. The *FN* rate was below 5 % for $n=200$ and $n=500$, and for $n=100$ with $\beta ^*=0.5$ and $\beta ^*=1$. The coverage of zero for $\beta _j= 0$ was close to 100 % for Algorithm 1 and around 95 % for Algorithm 2 for all sample and effect sizes. The coverage of zero for $\beta ^*$ was 0 % for both algorithms for $n=500$ for all effect sizes (Fig. 2). The coverage of ${\varvec{\beta }}^*$ for $\hat{\beta } \ne 0$ estimates was slightly lower for Algorithm 1 than 2. Algorithm 2 had 95 % coverage for $n=500$ and slightly below 95 % for $n=200$ (Fig. 3). Algorithm 1 had somewhat higher, albeit still less than 95 % coverage for the block correlation structure than the other correlations.

*Elastic net*

The *FP* rate ranged from 30.6 to 47.4 % for independent predictors, while it was below 4 % for the block correlation structure for all values of *n* and $\beta ^*$ (Table 4). For the AR correlation structure the *FP* rate was less than 5 % for $n=500$ for all values of $\beta ^*$. The *FN* rate was low for all correlation structures, and less than 5 % for $n=200$ and 500,  regardless of the effect sizes (Fig. 1 and Supplemental Table 3). The coverage of the zero coefficients was close to 100 % for both algorithms. The coverage of zero for coefficients corresponding to $\beta ^*$ was 0 % for both algorithms for $n=500$ for all effect sizes (Fig. 2). Overall, the coverage of $\beta ^*$ for $\hat{\beta } \ne 0$ coefficients was noticeably higher for Algorithm 2 than for Algorithm 1. Algorithm 2 had close to 95 % coverage with the exception of small sample sizes. For $n=100$ with $\beta ^*=0.25$ the coverage fell below 90 %, likely due to variables not being selected (Fig. 3).

*Relaxo*

The *FP* and *FN* rates were slightly lower for the block correlation structure than the independent or AR correlations, but were less than 5 % for $n=200$ and $n=500$ for all effect sizes and correlations (Fig. 1 and Supplemental Table 4). Both *FP* and *FN* rates also dropped quickly as *n* increased. For example, for the independent correlation structure and $\beta ^*=0.25$ the *FP* and *FN* rates were 15.6 and 33.0 % for $n=100$ and 3.1 and 0.4 % for $n=500$, respectively. For all correlation structures the coverage of zero for $\beta = 0$ was close to 100 % for both algorithms. The coverage of zero for $\beta ^*$ was close to 0 % for both algorithms for $n=500$ for all effect sizes (Fig. 2). The coverage of $\beta ^*$ for $\hat{\beta } \ne 0$ coefficients was similar for Algorithms 1 and 2, and close to 95 % for $n=500$ for all effect sizes, and for $n=200$ with $\beta ^*=0.5$ and $\beta ^*=1.0$ for all correlation structures (Fig. 3).

*SCAD*

The *FP* rates were very similar for all correlation structures and less than 5 % for $n=500$ with $\beta ^*=1$. The *FN* rate was generally low and dropped quickly as *n* increased (Fig. 1 and Supplemental Table 5). For $n=200$ or $n=500$ it was less than 5 % for all values of $\beta ^*$. It was greater than 10 % only for $n=100$ with $\beta ^*=0.25$. The coverage of zero for $\beta = 0$ was close to 100 % for Algorithm 1 and 95 % for Algorithm 2. The coverage of zero for $\beta ^*$ was 0 % for both algorithms for $n=500$ for all effect sizes (Fig. 2). For all correlation structures the coverage of the 95 % CIs of $\beta ^*$ for $\hat{\beta } \ne 0$ coefficients was close to 95 % for $n=500$ for Algorithm 2 for all effect sizes, and for Algorithm 1 for $n=500$ with $\beta ^*=1$. Again, the coverage was noticeably higher for Algorithm 2 than for Algorithm 1 and it increased for both algorithms with sample and effect size (Fig. 3).

*Penalized penalized linear regression*

The *FP* rate ranged from 17.9 % for $n=100$ with $\beta ^*=0.5$ and the block correlation structure to 48.5 % for $n=100$ with $\beta ^*=0.25$ and independent predictors (Fig. 1 and Supplemental Table 6). The *FN* rate was 16.2 % for $n=100$, $\beta ^*=0.25$ for the independent correlation structure, but for all other *n* and effect sizes it was less than 1 %. For all correlation structures the coverage of zero for the $\beta =0$ coefficients for Algorithm 1 was higher than 99 % for all sample sizes and effect sizes, while for Algorithm 2 the coverage was around 95 %. The coverage of zero for $\beta ^*$ was 0 % for both algorithms for $n=500$ for all values of $\beta ^*$. For independent $\mathbf{X}$, the coverage of $\beta ^*$ of the 95 % CIs for $\hat{\beta } \ne 0$ coefficients ranged from 78.7 to 86.6 % for Algorithm 1. It was around 95 % for Algorithm 2 only for $n=500$, but lower for $n=100$ and $n=200$. Similar patterns were seen for the AR correlation structure. For the block correlation structure both algorithms had close to 96 % coverage for $n=200$ and $n=500$ for all effect sizes (Fig. 3).

**Table 2:** Performance of the various methods when the error distribution in the linear model (1) was a *t*-distribution with two degrees of freedom for $\Sigma _X$ set to the identity matrix

Algorithm	*n*	$\beta ^*$	*FP* rate	*FN* rate	Coverage of zero for				Coverage of 95 % CIs of $\beta ^*$ for $\hat{\beta } \ne 0$	
					$\beta =0$		$\beta ^*\ne 0$			
					(Adapt$^\mathrm{a}$)	(Oracle$^\mathrm{b}$)	(Adapt)	(Oracle)	(Adapt)	(Oracle)
LARS	100	0.25	0.07	0.81	1.00	0.97	0.99	0.88	0.19	0.16
LARS	100	0.5	0.17	0.40	1.00	0.95	0.88	0.56	0.56	0.56
LARS	200	0.25	0.11	0.65	1.00	0.96	0.96	0.77	0.35	0.32
LARS	200	0.5	0.27	0.15	1.00	0.94	0.60	0.29	0.74	0.80
LARS	500	0.25	0.22	0.29	1.00	0.95	0.82	0.48	0.66	0.69
LARS	500	0.5	0.31	0.05	1.00	0.95	0.22	0.09	0.80	0.90
LASSO	100	0.25	0.07	0.81	1.00	0.97	0.99	0.88	0.19	0.16
LASSO	100	0.5	0.17	0.40	1.00	0.95	0.88	0.56	0.56	0.56
LASSO	200	0.25	0.11	0.65	1.00	0.96	0.96	0.77	0.35	0.32
LASSO	200	0.5	0.27	0.15	1.00	0.94	0.60	0.29	0.74	0.80
LASSO	500	0.25	0.22	0.29	1.00	0.95	0.82	0.48	0.66	0.68
LASSO	500	0.5	0.31	0.04	1.00	0.95	0.22	0.09	0.81	0.90
Elastic net	100	0.25	0.09	0.79	1.00	0.98	0.99	0.90	0.21	0.18
Elastic net	100	0.5	0.25	0.35	1.00	0.96	0.87	0.59	0.62	0.62
Elastic net	200	0.25	0.14	0.63	1.00	0.97	0.96	0.79	0.37	0.35
Elastic net	200	0.5	0.32	0.15	1.00	0.95	0.60	0.31	0.74	0.81
Elastic net	500	0.25	0.24	0.28	1.00	0.95	0.81	0.49	0.67	0.69
Elastic net	500	0.5	0.34	0.04	0.99	0.95	0.23	0.09	0.79	0.91
Relaxo	100	0.25	0.06	0.84	1.00	0.97	0.97	0.89	0.16	0.13
Relaxo	100	0.5	0.13	0.46	0.99	0.96	0.77	0.58	0.52	0.50
Relaxo	200	0.25	0.08	0.70	1.00	0.97	0.93	0.79	0.29	0.27
Relaxo	200	0.5	0.16	0.23	0.99	0.95	0.48	0.32	0.73	0.73
Relaxo	500	0.25	0.14	0.38	0.99	0.96	0.75	0.50	0.60	0.59
Relaxo	500	0.5	0.09	0.08	0.99	0.97	0.17	0.10	0.87	0.88
penalized.pls	100	0.25	0.01	0.93	0.99	0.99	0.95	0.95	0.06	0.06
penalized.pls	100	0.5	0.00	0.89	1.00	1.00	0.90	0.90	0.10	0.10
penalized.pls	200	0.25	0.00	0.92	1.00	1.00	0.93	0.93	0.07	0.07
penalized.pls	200	0.5	0.00	0.88	1.00	1.00	0.88	0.88	0.11	0.11
penalized.pls	500	0.25	0.00	0.90	1.00	1.00	0.91	0.91	0.09	0.09
penalized.pls	500	0.5	0.00	0.86	1.00	1.00	0.86	0.86	0.13	0.13
SCAD	100	0.25	0.97	0.02	1.00	0.95	0.99	0.86	0.98	0.93
SCAD	100	0.5	0.79	0.05	1.00	0.95	0.87	0.61	0.93	0.90
SCAD	200	0.25	1.00	0.00	0.98	0.95	0.81	0.75	0.98	0.95
SCAD	200	0.5	0.97	0.00	0.97	0.95	0.42	0.32	0.96	0.94
SCAD	500	0.25	1.00	0.00	0.95	0.95	0.49	0.48	0.95	0.95
SCAD	500	0.5	1.00	0.00	0.93	0.95	0.10	0.09	0.93	0.95

*FP* false positive, *FN* false negative

$^\mathrm{a}$ Corresponds to Algorithm 1, and $^\mathrm{b}$ to Algorithm 2 in the text

*Partial least squares*

The *FP* rate was less than 5 % for most correlation structures and effect sizes, the only outlier was the *FP* value of 15.3 % for $n=500$ and $\beta ^*=1$ with the independent correlation structure (Fig. 1 and Supplemental Table 7). However, the *FN* rate ranged from 15.3 % for $n=500$, $\beta ^*=1$ and the AR correlation structure to 87.6 % for $n=100$ with $\beta ^*=0.25$ for independent predictors, and was above 60 % for all correlation structures and most sample sizes. The *FN* rate was lower for larger effect and sample sizes. The coverage of zero for $\beta _j=0$ was around 100 % for both algorithms for settings. The coverage of zero for $\beta ^*$ for $n=500$ ranged from 15.3 to 81.2 %, and was not different for the two algorithms (Fig. 2). For both algorithms the coverage of $\beta ^*$ of the 95 % CIs for $\hat{\beta } \ne 0$ was very low for all correlation structures, ranging from 0 to $65.7\,\%$ (for independent $\mathbf{X}$, with $n=500$ and $\beta ^*=1$) (Fig. 3).

#### Non-normal error distribution

When we generated outcome data from a linear model (1) where the error term $\epsilon$ followed a t-distribution with 2 degrees of freedom for independent $\mathbf{X}$ (Table 2), the *FP* rate was lower for LARS, LASSO, elastic net and relaxo while for these methods the *FN* rate was higher compared to normally distributed errors. In contrast, for SCAD, the *FP* rate was much higher and the *FN* rate much lower than for normal errors. Simulation runs based on penalized partial least squares regression failed to give reasonable results in so many instances that we do not present any results for this method.

For all methods the coverage of zero for the $\beta =0$ coefficients for Algorithms 1 and 2 was very similar to the normal case. For all methods the coverage of zero for $\beta ^*$ was much higher than for normally distributed errors. The coverage of $\beta ^*$ of the 95 % CIs for $\hat{\beta } \ne 0$ coefficients however was much lower than in the normal case for Algorithm 1 and Algorithm 2 and much below the nominal 95 %. When the errors were generated from a t-distribution with 15 degrees of freedom however (Supplemental Table 8), the coverage was much improved and similar to that seen for normally distributed error terms.

#### Results for $p>n$

We also attempted to assess the performance of the methods when $$p>n$$ by generating data with $$ p=500$$ and $$p^*=10$$ for $$n=100, 200$$ and $$n=500$$ for independent predictors $$\mathbf{X}$$. SCAD, elastic net and penalized linear regression resulted in so many error messages that we do not present any findings for these algorithms.

Results for LARS, LASSO and relaxo are given in Table 3. For both LARS and LASSO, the *FP* rate was lower than for the $p<n$ setting for $n=100$ and 200, but was above 74 % for $n=500$ for all values of $\beta ^*$ The *FN* rate was low except for $\beta ^*=0.25$ with $n=100$. The coverage of zero for the $\beta =0$ coefficients for Algorithm 1 was higher than 99 % for all sample sizes and effect sizes, while for Algorithm 2 the coverage was below 70 %. The coverage of zero for $\beta ^*$ ranged from 0 to 93 % for Algorithm 1 and from 0 to 26 % for Algorithm 2. The coverage of $\beta ^*$ of the 95 % CIs for $\hat{\beta } \ne 0$ coefficients was less than 9 % for both algorithms.

For relaxo, the *FP* and *FN* rates were similar to those seen for LARS and LASSO for $n=100$ and $n=200$, with the exception of the *FP* rates for $n=500$, which were less than 2 %. The coverage of zero for the $\beta =0$ coefficients for Algorithm 1 ranged from 2 to 83 %, for all sample sizes and effect sizes, while for Algorithm 2 the coverage was below 62 %. The coverage of zero for $\beta ^*$ was below 5 % for $\beta ^*=0.5$ and $\beta ^*=1$ for both algorithms. The coverage of $\beta ^*$ of the 95 % CIs for $\hat{\beta } \ne 0$ coefficients was less than 10 % for both Algorithm 1 and 2.

**Table 3:** Results for $p=500$ and $p^*=10$ with the independent covariance matrix for linear regression models

Algorithm	*n*	$\beta ^*$	*FP* rate	*FN* rate	Coverage of zero for				Coverage of 95 % CIs of $\beta ^*$ for $\hat{\beta } \ne 0$	
					$\beta =0$		$\beta ^*\ne 0$			
					(Adapt$^\mathrm{a}$)	(Oracle$^\mathrm{b}$)	(Adapt)	(Oracle)	(Adapt)	(Oracle)
LARS	100	0.25	0.03	0.66	1	0.43	0.93	0.26	0.09	0.08
	200	0.25	0.09	0.13	1	0.55	0.71	0.15	0.06	0.07
	500	0.25	0.74	0	0.99	0.73	0.31	0.19	0.1	0.07
	100	0.5	0.08	0.09	1	0.59	0.63	0.1	0.06	0.04
	200	0.5	0.14	0	1	0.62	0.02	0	0.07	0.06
	500	0.5	0.88	0	0.99	0.71	0.28	0.15	0.1	0.07
	100	1	0.11	0	1	0.59	0.06	0	0.07	0.03
	200	1	0.16	0	0.99	0.63	0.01	0	0.07	0.06
	500	1	0.95	0	1	0.64	0.35	0.13	0.1	0.06
LASSO	100	0.25	0.03	0.65	0.99	0.43	0.93	0.26	0.09	0.08
	200	0.25	0.08	0.14	1	0.55	0.7	0.15	0.06	0.07
	500	0.25	0.79	0	0.97	0.75	0.29	0.22	0.09	0.08
	100	0.5	0.08	0.08	0.99	0.58	0.57	0.1	0.06	0.04
	200	0.5	0.13	0	0.99	0.63	0.01	0	0.07	0.06
	500	0.5	0.9	0	0.96	0.75	0.2	0.15	0.1	0.08
	100	1	0.11	0	0.98	0.58	0.02	0	0.07	0.03
	200	1	0.15	0	0.99	0.64	0	0	0.07	0.06
	500	1	0.96	0	0.96	0.71	0.23	0.13	0.1	0.07
Relaxo	100	0.25	0.02	0.76	0.83	0.26	0.64	0.14	0.09	0.08
	200	0.25	0.03	0.31	0.83	0.32	0.39	0.05	0.08	0.09
	500	0.25	0.01	0.02	0.2	0.05	0	0	0.09	0.09
	100	0.5	0.04	0.2	0.74	0.48	0.2	0.05	0.06	0.07
	200	0.5	0.01	0.01	0.38	0.33	0	0	0.09	0.09
	500	0.5	0	0	0.02	0.02	0	0	0.09	0.09
	100	1	0.03	0	0.63	0.62	0	0	0.07	0.07
	200	1	0	0	0.35	0.34	0	0	0.09	0.09
	500	1	0	0	0.02	0.01	0	0	0.09	0.09

**Table 4:** LASSO for logistic regression models based on case-control data with $n_1$ cases and $n_0$ controls

$F_{\mathbf {X}}$	*n*	$\beta ^*$	*FP* rate	*FN* rate	Coverage of zero for				Coverage of 95 % CIs of $\beta ^*$ for $\hat{\beta } \ne 0$	
					$\beta =0$		$\beta ^*\ne 0$			
					(Adapt$^\mathrm{a}$)	(Oracle$^\mathrm{b}$)	(Adapt)	(Oracle)	(Adapt)	(Oracle)
Normal	100	0.25	0.069	0.814	0.999	0.970	0.997	0.945	0.104	0.073
Normal	200	0.25	0.080	0.776	0.998	0.968	0.994	0.917	0.146	0.116
Normal	500	0.25	0.140	0.593	0.998	0.962	0.975	0.804	0.346	0.319
Normal	100	0.500	0.102	0.688	0.999	0.963	0.988	0.860	0.242	0.202
Normal	200	0.500	0.198	0.423	0.997	0.951	0.930	0.677	0.532	0.489
Normal	500	0.500	0.365	0.063	0.994	0.946	0.634	0.287	0.828	0.896
Normal	100	1.000	0.262	0.229	0.997	0.934	0.853	0.488	0.700	0.668
Normal	200	1.00	0.385	0.022	0.994	0.936	0.488	0.166	0.832	0.912
Normal	500	1.00	0.443	0.000	0.993	0.945	0.029	0.004	0.850	0.937
Binomial	100	0.25	0.086	0.760	0.999	0.965	0.992	0.907	0.163	0.125
Binomial	200	0.25	0.120	0.646	0.998	0.960	0.976	0.828	0.288	0.25
Binomial	500	0.25	0.264	0.259	0.996	0.949	0.859	0.533	0.713	0.683
Binomial	100	0.50	0.177	0.451	0.998	0.947	0.941	0.683	0.502	0.445
Binomial	200	0.50	0.314	0.119	0.996	0.938	0.750	0.381	0.778	0.817
Binomial	500	0.50	0.425	0.002	0.994	0.944	0.192	0.046	0.859	0.940
Binomial	100	1.00	0.367	0.039	0.996	0.915	0.578	0.206	0.793	0.815
Binomial	200	1.00	0.442	0.001	0.994	0.931	0.121	0.020	0.824	0.897
Binomial	500	1.00	0.483	0.000	0.993	0.942	0.000	0.000	0.813	0.930

$F_X$ is the distribution of the predictors $\mathbf{X}$

*FP* false positive, *FN* false negative

$^\mathrm{a}$ Corresponds to Algorithm 1, and $^\mathrm{b}$ to Algorithm 2 in the text

#### Summary of results for linear regression

The estimation methods that had a high false positive (FP) rate were LARS, LASSO, elastic net, SCAD and penalized linear regression. Not surprisingly, the FN rate of these methods was low. Partial least squares regression had a low FP rate at the cost of having many false negatives. Only relaxo had both a low FP and FN rate. The coverage of zero for the $\beta =0$ coefficients for Algorithm 1 was close to 100 % for all methods, while for Algorithm 2 it was closer to 95 %. The coverage of zero of the $\beta ^*$ coefficients was close to zero for all methods with the exception of penalized least squares (Fig. 3). The coverage of the true $\beta ^*$ coefficients of the 95 % CIs around $\hat{\beta } \ne 0$ was typically higher for Algorithm 2 than for Algorithm 1. For Algorithm 2 it was close to 95 % for large sample sizes and effect sizes for all methods with the exception of penalized partial least squares, for which coverage even for $n=500$ with $\beta ^*=1$ was around 65 %. When $p>n$, the coverage of both algorithms was much lower than 95 %, however.

### Results for logistic regression

*LASSO*

The *FP* rate was somewhat higher for binary predictors than for independent normally distributed $\mathbf{X}$, but for both it was appreciable, with values up to 44 % even for large effect and sample sizes (Table 4). The *FN* rate was above 50 % for $\beta ^*=0.25$, but was less than 6 % for binary predictors with $\beta ^*=1.0$ for all sample sizes, for $\beta ^*=0.5$ for $n=200$ and 500, and for normally distributed $\mathbf{X}$ with $\beta ^*=1.0$ for $n=200$ and 500. The coverage of zero for $\beta = 0$ was nearly 100 % for Algorithm 1 and closer to 95 % for Algorithm 2. The coverage of zero for $\beta ^*$ was higher for Algorithm 1 than Algorithm 2. For Algorithm 1 the coverage of zero for $\beta ^*$ ranged from 0 % for $n=500$ with $\beta ^*=1$ and binary predictors to 99.7 % for $n=100$ with $\beta ^*=0.25$. The coverage of $\beta ^*$ of the 95 % CIs around $\hat{\beta } \ne 0$ was very low for both algorithms, with the exception of $n=500$ and $\beta ^*=1.0$ for normally distributed and $\beta ^*=0.5$ and $\beta ^*=1.0$ for binary $\mathbf{X}$, where coverage was close to 95 %.

*SCAD*

For both, independent normally distributed and binary $\mathbf{X}$ the *FP* rate was very low; the largest value was 5 % for $n=500$ with $\beta ^*=0.5$, while the *FN* rate was extremely high, with values above 80 % for many other settings (Table 5). Only for $n=500$ with $\beta ^*=1.0$ and for binary predictors also for $\beta ^*=0.5$ was the *FN* rate below 15 %. The coverage of zero for $\hat{\beta }_j= 0$ was nearly 100 % for both algorithms. The coverage of zero for $\beta ^*\ne 0$ was similar for both algorithms and ranged from 0.04 to 99.6 %. It dropped as sample size and effect size increased. The coverage of $\beta ^*$ of the 95 % CIs around $\hat{\beta } \ne 0$ was very low for both algorithms, with the exception of $n=500$ and $\beta ^*=1.0$ for both normally distributed and binary predictors, for which the coverage was approximately 93 %.

*Penalized logistic regression*

For the independent normally distributed and binary predictors $\mathbf{X}$ the *FP* rate was similar, and ranged from 44.2 to 72.0 %. We observed an *FP* of 45.3 % for independent normal $\mathbf{X}$ even for $\beta ^*=1$ and $n=500$ (Table 6). The *FN* rate depended more strongly on the effect size, was somewhat higher for normally distributed $\mathbf{X}$ but in all cases decreased noticeably as *n* increased. For example, for normally distributed predictors with $\beta ^*=0.5$, the *FN* rate was 28.3 % for $n=100$, 13.4 % for $n=200$ and 3 % for $n=500$. The coverage of zero for $\beta = 0$ was nearly 100 % for Algorithm 1 and between 91.5 and 94.8 % for Algorithm 2. The coverage of zero for $\beta ^*$ ranged from from 0.0 to 99.9 % for Algorithm 1 and was slightly lower for Algorithm 2. It dropped as sample size and effect size increased for both algorithms. The coverage of $\beta ^*$ of the 95 % CIs around $\hat{\beta } \ne 0$ was slightly lower for Algorithm 1 than 2. For $\beta ^*=0.5$ and $\beta ^*=1.0$ with $n=500$ Algorithm 2 had a coverage of nearly 95 %.

**Table 5:** SCAD for logistic regression models based on case-control data with $n_1$ cases and $n_0$ controls

$F_{\mathbf {X}}$	*n*	$\beta ^*$	*FP* rate	*FN* rate	Coverage of zero for				Coverage of 95 % CIs of $\beta ^*$ for $\hat{\beta } \ne 0$	
					$\beta =0$		$\beta ^*\ne 0$			
					(Adapt$^\mathrm{a}$)	(Oracle$^\mathrm{b}$)	(Adapt)	(Oracle)	(Adapt)	(Oracle)
Normal	100	0.25	0.002	0.995	0.999	0.998	0.996	0.995	0.004	0.001
Normal	200	0.25	0.003	0.992	0.998	0.997	0.992	0.992	0.005	0.002
Normal	500	0.25	0.004	0.979	0.997	0.997	0.980	0.979	0.015	0.013
Normal	100	0.500	0.002	0.986	0.999	0.998	0.989	0.985	0.014	0.007
Normal	200	0.500	0.007	0.938	0.995	0.994	0.937	0.933	0.061	0.047
Normal	500	0.500	0.052	0.526	0.971	0.968	0.525	0.514	0.503	0.495
Normal	100	1.000	0.005	0.905	0.999	0.996	0.937	0.899	0.104	0.093
Normal	200	1.000	0.031	0.422	0.982	0.977	0.399	0.377	0.624	0.610
Normal	500	1.000	0.024	0.137	0.987	0.986	0.055	0.051	0.924	0.925
Binomial	100	0.25	0.002	0.984	0.999	0.999	0.996	0.994	0.006	0.003
Binomial	200	0.25	0.002	0.977	0.999	0.999	0.985	0.982	0.015	0.011
Binomial	500	0.25	0.005	0.858	0.996	0.995	0.884	0.867	0.119	0.112
Binomial	100	0.500	0.002	0.945	0.999	0.998	0.979	0.965	0.037	0.029
Binomial	200	0.500	0.015	0.732	0.992	0.990	0.778	0.751	0.249	0.231
Binomial	500	0.500	0.018	0.148	0.987	0.983	0.133	0.110	0.850	0.857
Binomial	100	1.000	0.004	0.796	0.999	0.998	0.891	0.823	0.135	0.169
Binomial	200	1.000	0.020	0.126	0.987	0.982	0.103	0.091	0.870	0.862
Binomial	500	1.000	0.014	0.057	0.990	0.988	0.004	0.003	0.940	0.937

$F_X$ is the distribution of the predictors $\mathbf{X}$

*FP* false positive, *FN* false negative

$^\mathrm{a}$ Corresponds to Algorithm 1, and $^\mathrm{b}$ to Algorithm 2 in the text

**Table 6:** Penalized logistic regression based on case-control data with $n_1$ cases and $n_0$ controls

$F_{\mathbf {X}}$	*n*	$\beta ^*$	*FP* rate	*FN* rate	Coverage of zero for				Coverage of 95 % CIs of $\beta ^*$ for $\hat{\beta } \ne 0$	
					$\beta =0$		$\beta ^*\ne 0$			
					(Adapt$^\mathrm{a}$)	(Oracle$^\mathrm{b}$)	(Adapt)	(Oracle)	(Adapt)	(Oracle)
Normal	100	0.25	0.442	0.492	0.999	0.944	0.999	0.914	0.459	0.395
Normal	200	0.25	0.511	0.423	1.000	0.948	0.998	0.878	0.558	0.512
Normal	500	0.25	0.626	0.212	0.999	0.948	0.986	0.746	0.765	0.716
Normal	100	0.5	0.629	0.283	1.000	0.925	0.992	0.800	0.688	0.611
Normal	200	0.5	0.706	0.134	0.999	0.936	0.973	0.641	0.852	0.795
Normal	500	0.5	0.634	0.030	0.997	0.945	0.743	0.293	0.853	0.926
Normal	100	1	0.616	0.103	1.000	0.922	0.949	0.470	0.822	0.790
Normal	200	1	0.453	0.017	0.998	0.937	0.606	0.169	0.764	0.918
Normal	500	1	0.461	0.000	0.992	0.944	0.035	0.003	0.835	0.934
Binomial	100	0.25	0.553	0.359	1.000	0.930	0.997	0.852	0.605	0.530
Binomial	200	0.25	0.578	0.278	0.999	0.941	0.990	0.776	0.703	0.650
Binomial	500	0.25	0.648	0.083	0.998	0.946	0.911	0.507	0.901	0.866
Binomial	100	0.5	0.720	0.115	0.999	0.912	0.971	0.613	0.872	0.773
Binomial	200	0.5	0.631	0.050	0.997	0.937	0.839	0.359	0.859	0.877
Binomial	500	0.5	0.548	0.002	0.993	0.942	0.212	0.045	0.844	0.941
Binomial	100	1	0.496	0.029	0.998	0.915	0.724	0.205	0.706	0.824
Binomial	200	1	0.486	0.001	0.995	0.933	0.133	0.024	0.802	0.894
Binomial	500	1	0.494	0.000	0.991	0.943	0.000	0.000	0.799	0.932

$F_X$ is the distribution of the predictors $\mathbf{X}$

*FP* false positive, *FN* false negative

$^\mathrm{a}$ Corresponds to Algorithm 1, and $^\mathrm{b}$ to Algorithm 2 in the text

**Table 7:** Adaptive logistic regression based on case-control data with $n_1$ cases and $n_0$ controls

Algorithm	*n*	$\beta ^*$	*FP* rate	*FN* rate	Coverage of zero for				Coverage of 95 % CIs of $\beta ^*$ for $\hat{\beta } \ne 0$	
					$\beta =0$		$\beta ^*\ne 0$			
					(Adapt$^\mathrm{a}$)	(Oracle$^\mathrm{b}$)	(Adapt)	(Oracle)	(Adapt)	(Oracle)
Normal	100	0.25	0.04	0.85	1	0.98	0.99	0.97	0.06	0.04
Normal	200	0.25	0.04	0.84	1	0.98	0.99	0.95	0.07	0.05
Normal	500	0.25	0.05	0.77	1	0.98	0.96	0.89	0.15	0.13
Normal	100	0.5	0.06	0.8	0.99	0.98	0.98	0.91	0.12	0.1
Normal	200	0.5	0.08	0.67	0.99	0.97	0.93	0.8	0.25	0.23
Normal	500	0.5	0.2	0.22	0.98	0.95	0.61	0.34	0.67	0.73
Normal	200	1	0.2	0.14	0.98	0.95	0.49	0.22	0.69	0.79
Normal	500	1	0.22	0	0.98	0.95	0.06	0.01	0.82	0.94
Binomial	100	0.25	0.06	0.81	0.99	0.98	0.98	0.93	0.1	0.08
Binomial	200	0.25	0.07	0.76	0.99	0.98	0.96	0.89	0.15	0.13
Binomial	500	0.25	0.12	0.51	0.99	0.97	0.84	0.65	0.43	0.41
Binomial	200	0.5	0.16	0.33	0.99	0.96	0.73	0.46	0.57	0.59
Binomial	500	0.5	0.23	0.03	0.98	0.95	0.22	0.05	0.81	0.93
Binomial	200	1	0.21	0.02	0.98	0.95	0.16	0.03	0.78	0.91
Binomial	500	1	0.16	0	0.99	0.96	0	0	0.82	0.94

*FP* false positive, *FN* false negative

$^\mathrm{a}$ Corresponds to Algorithm 1, and $^\mathrm{b}$ to Algorithm 2 in the text

*Adaptive logistic regression*

For all $\mathbf{X}$ the *FP* rate was less than 10 % for $n=200$ and $n=100$ for all effect sizes, while the *FN* rates for those *n* ranged from 67 to 85 % (Table 7). For $n=500$, the *FP* rate was 5 % for $\beta ^*=0.25$ and 20 and 22 % for $\beta ^*=0.5$ and $\beta ^*=1$ respectively, with corresponding *FN* rates of 77, 22 and 0. %. For binary predictors the *FP* rate was higher, and ranged from 6 to 23 %, and *FN* rates ranged from 0 to 81 %. The coverage of zero for $\beta = 0$ was nearly 100 % for Algorithm 1 for sample sizes $n=100$ and $n=200$. For $n=500$ with effect sizes $\beta ^*=0.5$ and $\beta ^*=1.0$ the coverage for Algorithm 1 was 95 %. The coverage of zero for $\beta ^*$ ranged from from 0.0 to 99 % for Algorithm 1 and was slightly lower for Algorithm 2. It dropped as sample size and effect size increased for both algorithms. The coverage of $\beta ^*$ of the 95 % CIs around $\hat{\beta } \ne 0$ was slightly lower for Algorithm 1 than 2. However, Algorithm 2 had 94 % coverage only for $n=500$ and $\beta ^*=1.0$ and $\beta ^*=0.5$. For all other sample and effect sizes coverage ranged from 4 to 93 % for Algorithm 2 and from 6 to 82 % for Algorithm 1.

#### Summary of results for logistic regression

LASSO and penalized logistic regression had a high FP rate and a low FN rate. SCAD had a low FP rate at the cost of having many FNs. Adaptive logistic regression had a moderate FP rate and a high FN rate. The coverage of zero for the $\beta =0$ coefficients was close to 100 % for Algorithm 1, while for Algorithm 2 it was closer to 95 % for all methods. The coverage of zero of the $\beta ^*$ coefficients was close to zero for all methods with the exception of penalized logistic regression. The coverage of the true $\beta ^*$ coefficients of the 95 % CIs around $\hat{\beta } \ne 0$ was close to 95 % for Algorithm 2 for large sample sizes and effect sizes for all methods with the exception of penalized logistic regression for which coverage even for $n=500$ with $\beta ^*=1$ was around 80 %. It was lower for Algorithm 1.

## Discussion

Penalized estimation methods deliberately introduce a bias to reduce variability of the estimates to identify outcome-associated variables, and have been typically applied to prediction. Nonetheless, penalized regression techniques are also used sometimes when the aim is inference. For example, they have been applied to molecular genetic data for both prediction, and identification of disease susceptibility genes. We therefore assessed the performance of several readily available penalized estimation methods for linear and logistic regression. We performed only a small simulation study for the setting of $p>n$ for which asymptotic results on consistent variable selection are very limited. Our main focus was on situations often encountered in practical settings, where the sample size *n* ranges from twofold larger to tenfold larger than the number of parameters, *p*.

First we quantified the methods’ ability to identify truly outcome associated predictors, i.e. to estimate the sparsity patterns of a vector ${\varvec{\beta }}$ of regression coefficients. For linear models, penalized linear regression, elastic net, smoothly clipped absolute deviation (SCAD), least angle regression (LARS) and LASSO had a low false negative (*FN*) predictor selection rates but false positive (*FP*) rates above 20 % for all sample and effect sizes. Partial least squares regression had few *FP*s but many *FN*s. Only relaxo had low *FP* and *FN* rates. For logistic models, LASSO and penalized logistic regression had many FPs and few *FN*s for all sample and effect sizes. SCAD and adaptive logistic regression had low or moderate *FP* rates but many *FN*s.

We also evaluated inference properties for the various procedures. We studied effect estimates obtained directly from penalized methods (Algorithm 1), or by refitting selected predictors with standard regression (Algorithm 2). 95 % confidence interval coverage of predictors with null effects was approximately 100 % for Algorithm 1 for all methods, and 95 % for Algorithm 2 for large sample and effect sizes. Coverage was low only for penalized partial least squares (linear regression). For outcome-associated predictors, coverage was close to 95 % for Algorithm 2 for large sample and effect sizes for all methods except penalized partial least squares and penalized logistic regression. Coverage was sub-nominal for Algorithm 1. In conclusion, while Algorithm 2 is preferred to Algorithm 1, estimates from Algorithm 2 are still prone to some bias arising from the selection of predictors, which affects those associated with moderate effect sizes more strongly than predictors with large effect sizes.

All procedures were somewhat sensitive to violations of the assumption of normality for the error distribution for the linear model. When we generated outcome data from a linear model where the error term $\epsilon$ followed a t-distribution with 2 degrees of freedom the *FN* rate was higher compared to normally distributed errors for LARS, LASSO, elastic net and relaxo, while for SCAD the *FP* rate was much higher, and penalized partial least squares regression generally failed to give results. For outcome-associated predictors, the coverage of the 95 % CIs was much below the nominal 95 % for all procedures.

We addressed the problem of coverage much more extensively than previous publications (e.g. Wang and Leng 2007; Kabaila and Leeb 2006), including many popular penalized methods in our simulations, and also focused on false positive and false negative findings. We simulated practically relevant settings that reflect the number of predictors seen in many datasets, and showed that even for large sample sizes estimates are subject to undue bias and variance from the model selection procedure. Refitting attenuates the bias, but does not eliminate it in all but the cases where there is large sample size combined with estimating large effects. In these settings the residual bias not compensated for in refitting was small enough to be negligible. In all other settings where data is limited or effect sizes are small, the bias and variance are large enough to invalidate inference after model selection on those parameters, even for Algorithm 2.

When simulations were based on $p>n$, SCAD, elastic net, and penalized linear regression (the implementations we used) resulted in so many error messages that it was not meaningful to present any findings for them. For LARS and LASSO the *FN* rate was low and the *FP* rate was lower than for the $p<n$ setting for moderate sample sizes but was above 74 % for $n=500$ for all values of $\beta ^*$. For relaxo, the *FP* and *FN* rates were similar to those seen for LARS and LASSO but low also for large *n*. The coverage of $\beta ^*$ of the 95 % CIs for $\hat{\beta } \ne 0$ coefficients was much below the nominal level for both Algorithm 1 and 2.

There is a growing literature on valid inference after model selection. E.g., Efron (2014), Wasserman and Roeder (2009) and Meinshausen et al. (2009) proposed approaches based on resampling or data splitting. Lockhart et al. (2014) derived the exact asymptotic null distribution of a test statistic for significance of variables that enter the LASSO model for general design matrices $\mathbf{X}$ and extends results to elastic net estimates. Berk et al. (2013) proposed an approach for post-selection inference (“PoSI”) that is valid over all possible selected models and does not assume the linear model is correct. A better understanding of the small sample properties of some of these techniques is still needed. Nonetheless translation of the above mentioned approaches and others into statistical practice is also important to avoid misleading inference and irreproducible scientific findings.

## Electronic supplementary material

Below is the link to the electronic supplementary material.

Supplementary material 1 (pdf 49 KB)
