# Supplementary material for: On the impact of model selection on predictor identification and parameter inference
Source: Comput Stat. 2016 Oct 22;32(2):667–90. doi: 10.1007/s00180-016-0690-2 (PMC5480098; doi:10.1007/s00180-016-0690-2)
Supplement: Supplementary file 1 — Supplementary material 1 (pdf 49 KB) [file 180_2016_690_MOESM1_ESM.pdf]

Supplemental tables for "On the impact of model selection on predictor identification and parameter inference", Computational Statistics

Ruth M. Pfeiffer[1], Andrew Redd[2], Raymond Carroll[3]

[1] National Cancer Institute, Division of Cancer Epidemiology and Genetics, 9609 Medical Center Drive, Bethesda, MD 20892
[2] Department of Internal Medicine, University of Utah School of Medicine, Salt Lake City, UT, 84132
[3] Department of Statistics, Texas A&M University, College Station, TX 77843-3143.

Corresponding author: Ruth M. Pfeiffer
National Cancer Institute
9609 Medical Center Drive, Room 7E142
Bethesda, MD 20892
email: pfeiffer@mail.nih.gov
Phone: (240) 276-7317

Table 1: Lars for linear regression

Σ_X	n	β^*	FP rate	FN rate	coverage of zero for $\beta = 0$ (adapt[1])	coverage of zero for $\beta = 0$ (oracle[2])	coverage of zero for $\beta^* \neq 0$ (adapt)	coverage of zero for $\beta^* \neq 0$ (oracle)	coverage of 95% CIs of β^* for $\hat{\beta} \neq 0$ (adapt)	coverage of 95% CIs of β^* for $\hat{\beta} \neq 0$ (oracle)
indep	100	0.25	0.220	0.232	0.997	0.940	0.830	0.449	0.715	0.729
indep	200	0.25	0.319	0.016	0.996	0.940	0.384	0.100	0.814	0.916
indep	500	0.25	0.344	0.000	0.995	0.947	0.007	0.001	0.829	0.939
indep	100	0.5	0.285	0.002	0.996	0.935	0.136	0.014	0.806	0.895
indep	200	0.5	0.322	0.000	0.996	0.940	0.000	0.000	0.825	0.920
indep	500	0.5	0.348	0.000	0.994	0.944	0.000	0.000	0.822	0.941
indep	100	1	0.254	0.000	0.997	0.937	0.000	0.000	0.778	0.891
indep	200	1	0.337	0.000	0.996	0.941	0.000	0.000	0.809	0.925
indep	500	1	0.451	0.000	0.990	0.946	0.000	0.000	0.833	0.940
block	100	0.25	0.111	0.108	0.999	0.963	0.763	0.534	0.883	0.843
block	200	0.25	0.148	0.013	0.999	0.958	0.399	0.244	0.934	0.935
block	500	0.25	0.157	0.000	0.998	0.959	0.032	0.012	0.934	0.945
block	100	0.5	0.106	0.003	0.999	0.965	0.198	0.063	0.927	0.922
block	200	0.5	0.156	0.000	0.999	0.957	0.003	0.001	0.931	0.935
block	500	0.5	0.147	0.000	0.999	0.958	0.000	0.000	0.931	0.943
block	100	1	0.110	0.000	0.999	0.966	0.001	0.000	0.923	0.925
block	200	1	0.157	0.000	0.998	0.963	0.000	0.000	0.918	0.940
block	500	1	0.194	0.000	0.997	0.969	0.000	0.000	0.911	0.947
AR	100	0.25	0.154	0.178	0.999	0.957	0.801	0.529	0.809	0.762
AR	200	0.25	0.210	0.043	0.998	0.951	0.477	0.258	0.868	0.897
AR	500	0.25	0.235	0.002	0.998	0.954	0.070	0.014	0.875	0.939
AR	100	0.5	0.178	0.020	0.998	0.955	0.259	0.080	0.865	0.895
AR	200	0.5	0.228	0.000	0.997	0.950	0.017	0.001	0.881	0.934
AR	500	0.5	0.240	0.000	0.997	0.949	0.000	0.000	0.877	0.936
AR	100	1	0.179	0.000	0.998	0.954	0.008	0.000	0.852	0.917
AR	200	1	0.218	0.000	0.997	0.953	0.000	0.000	0.860	0.932
AR	500	1	0.202	0.000	0.997	0.959	0.000	0.000	0.834	0.944

FP - false positive; FN - false negative

[1] corresponds to Algorithm 1, and [2] to Algorithm 2 in the text

Σ_X is the covariance matrix of the predictors $\mathbf{X}$

Table 2: LASSO for linear regression

Σ_X	n	β^*	FP rate	FN rate	coverage of zero for $\beta = 0$ (adapt[1])	(oracle[2])	$\beta^* \neq 0$ (adapt)	(oracle)	coverage of 95% CIs of β^* for $\hat{\beta} \neq 0$ (adapt)	(oracle)
indep	100	0.25	0.217	0.240	0.997	0.943	0.837	0.457	0.712	0.718
indep	200	0.25	0.314	0.016	0.996	0.942	0.384	0.097	0.818	0.920
indep	500	0.25	0.338	0.000	0.996	0.946	0.007	0.000	0.828	0.938
indep	100	0.5	0.291	0.001	0.995	0.934	0.143	0.015	0.804	0.893
indep	200	0.5	0.322	0.000	0.996	0.940	0.000	0.000	0.827	0.925
indep	500	0.5	0.334	0.000	0.995	0.945	0.000	0.000	0.812	0.939
indep	100	1	0.257	0.000	0.997	0.939	0.000	0.000	0.779	0.902
indep	200	1	0.345	0.000	0.995	0.942	0.000	0.000	0.826	0.930
indep	500	1	0.446	0.000	0.992	0.946	0.000	0.000	0.836	0.942
block	100	0.25	0.120	0.107	0.999	0.962	0.758	0.534	0.884	0.847
block	200	0.25	0.153	0.014	0.998	0.959	0.398	0.247	0.937	0.932
block	500	0.25	0.152	0.000	0.999	0.959	0.032	0.011	0.933	0.947
block	100	0.5	0.103	0.003	0.999	0.966	0.193	0.063	0.927	0.922
block	200	0.5	0.157	0.000	0.999	0.957	0.006	0.002	0.936	0.933
block	500	0.5	0.153	0.000	0.998	0.958	0.000	0.000	0.927	0.943
block	100	1	0.103	0.000	0.999	0.969	0.002	0.000	0.923	0.929
block	200	1	0.159	0.000	0.998	0.963	0.000	0.000	0.919	0.935
block	500	1	0.199	0.000	0.997	0.969	0.000	0.000	0.913	0.945
AR	100	0.25	0.160	0.184	0.998	0.956	0.795	0.527	0.805	0.762
AR	200	0.25	0.212	0.044	0.998	0.954	0.471	0.253	0.875	0.896
AR	500	0.25	0.241	0.001	0.998	0.951	0.061	0.011	0.883	0.944
AR	100	0.5	0.182	0.020	0.998	0.951	0.255	0.082	0.860	0.889
AR	200	0.5	0.238	0.000	0.997	0.950	0.020	0.001	0.883	0.931
AR	500	0.5	0.239	0.000	0.998	0.949	0.000	0.000	0.878	0.941
AR	100	1	0.171	0.000	0.998	0.955	0.007	0.000	0.845	0.909
AR	200	1	0.208	0.000	0.997	0.954	0.000	0.000	0.854	0.928
AR	500	1	0.189	0.000	0.997	0.958	0.000	0.000	0.830	0.944

FP - false positive; FN - false negative

[1] corresponds to Algorithm 1, and [2] to Algorithm 2 in the text

Σ_X is the covariance matrix of the predictors $\mathbf{X}$

Table 3: Elastic net for linear regression

Σ_X	n	β^*	FP rate	FN rate	coverage of zero for $\beta = 0$ (adapt[1])	(oracle[2])	$\beta^* \neq 0$ (adapt)	(oracle)	coverage of 95% CIs of β^* for $\hat{\beta} \neq 0$ (adapt)	(oracle)
indep	100	0.25	0.306	0.211	0.997	0.953	0.832	0.505	0.752	0.761
indep	200	0.25	0.384	0.017	0.994	0.948	0.387	0.110	0.830	0.932
indep	500	0.25	0.373	0.000	0.993	0.951	0.012	0.000	0.813	0.944
indep	100	0.5	0.460	0.005	0.991	0.957	0.235	0.025	0.814	0.930
indep	200	0.5	0.420	0.000	0.984	0.952	0.006	0.000	0.739	0.944
indep	500	0.5	0.367	0.000	0.983	0.956	0.000	0.000	0.705	0.947
indep	100	1	0.474	0.000	0.975	0.966	0.017	0.000	0.669	0.941
indep	200	1	0.415	0.000	0.957	0.967	0.000	0.000	0.552	0.941
indep	500	1	0.413	0.000	0.945	0.968	0.000	0.000	0.528	0.951
block	100	0.25	0.044	0.014	1.000	0.988	0.623	0.562	0.982	0.939
block	200	0.25	0.004	0.001	1.000	0.998	0.255	0.247	0.984	0.947
block	500	0.25	0.000	0.000	1.000	1.000	0.002	0.010	0.985	0.950
block	100	0.5	0.011	0.000	1.000	0.997	0.060	0.057	0.967	0.946
block	200	0.5	0.000	0.000	1.000	1.000	0.001	0.001	0.954	0.951
block	500	0.5	0.000	0.000	1.000	1.000	0.000	0.000	0.894	0.946
block	100	1	0.001	0.000	1.000	1.000	0.002	0.000	0.854	0.951
block	200	1	0.000	0.000	1.000	1.000	0.000	0.000	0.783	0.951
block	500	1	0.000	0.000	1.000	1.000	0.000	0.000	0.789	0.950
AR	100	0.25	0.175	0.093	1.000	0.973	0.743	0.583	0.902	0.857
AR	200	0.25	0.111	0.052	1.000	0.977	0.348	0.273	0.882	0.895
AR	500	0.25	0.036	0.010	1.000	0.988	0.159	0.021	0.798	0.935
AR	100	0.5	0.129	0.031	0.999	0.982	0.212	0.093	0.855	0.908
AR	200	0.5	0.057	0.013	0.999	0.990	0.126	0.014	0.770	0.922
AR	500	0.5	0.019	0.001	0.998	0.998	0.026	0.001	0.661	0.948
AR	100	1	0.107	0.017	0.996	0.990	0.100	0.017	0.752	0.915
AR	200	1	0.034	0.005	0.997	0.997	0.034	0.005	0.619	0.934
AR	500	1	0.012	0.000	0.998	0.999	0.009	0.000	0.483	0.948

FP - false positive; FN - false negative

[1] corresponds to Algorithm 1, and [2] to Algorithm 2 in the text

Σ_X is the covariance matrix of the predictors $\mathbf{X}$

Table 4: Relaxo for linear regression

Σ_X	n	β^*	FP rate	FN rate	coverage of zero for $\beta = 0$ (adapt[1])	(oracle[2])	$\beta^* \neq 0$ (adapt)	(oracle)	coverage of 95% CIs of β^* for $\hat{\beta} \neq 0$ (adapt)	(oracle)
indep	100	0.25	0.156	0.330	0.989	0.953	0.701	0.488	0.647	0.636
indep	200	0.25	0.179	0.067	0.985	0.950	0.235	0.118	0.859	0.891
indep	500	0.25	0.031	0.004	0.986	0.981	0.004	0.004	0.938	0.945
indep	100	0.5	0.167	0.015	0.975	0.953	0.041	0.023	0.880	0.898
indep	200	0.5	0.040	0.000	0.984	0.980	0.000	0.000	0.938	0.940
indep	500	0.5	0.011	0.000	0.993	0.992	0.000	0.000	0.950	0.951
indep	100	1	0.071	0.000	0.979	0.975	0.000	0.000	0.926	0.928
indep	200	1	0.014	0.000	0.993	0.992	0.000	0.000	0.945	0.945
indep	500	1	0.014	0.000	0.992	0.991	0.000	0.000	0.944	0.945
block	100	0.25	0.048	0.184	0.992	0.981	0.565	0.512	0.775	0.762
block	200	0.25	0.033	0.056	0.993	0.986	0.269	0.246	0.904	0.902
block	500	0.25	0.019	0.003	0.994	0.990	0.013	0.013	0.946	0.947
block	100	0.5	0.031	0.013	0.992	0.987	0.067	0.063	0.932	0.930
block	200	0.5	0.020	0.001	0.994	0.990	0.001	0.001	0.945	0.944
block	500	0.5	0.019	0.000	0.994	0.990	0.000	0.000	0.952	0.952
block	100	1	0.022	0.000	0.994	0.990	0.000	0.000	0.946	0.945
block	200	1	0.020	0.000	0.994	0.990	0.000	0.000	0.944	0.943
block	500	1	0.020	0.000	0.994	0.990	0.000	0.000	0.948	0.948
AR	100	0.25	0.094	0.298	0.990	0.970	0.632	0.554	0.659	0.634
AR	200	0.25	0.081	0.142	0.991	0.973	0.346	0.296	0.805	0.798
AR	500	0.25	0.061	0.033	0.988	0.976	0.043	0.040	0.908	0.910
AR	100	0.5	0.092	0.065	0.987	0.972	0.131	0.113	0.852	0.850
AR	200	0.5	0.051	0.009	0.988	0.979	0.010	0.009	0.920	0.928
AR	500	0.5	0.018	0.000	0.993	0.990	0.000	0.000	0.948	0.949
AR	100	1	0.055	0.001	0.985	0.980	0.001	0.001	0.927	0.930
AR	200	1	0.018	0.000	0.993	0.991	0.000	0.000	0.943	0.944
AR	500	1	0.019	0.000	0.993	0.990	0.000	0.000	0.945	0.947

FP - false positive; FN - false negative

[1] corresponds to Algorithm 1, and [2] to Algorithm 2 in the text

Σ_X is the covariance matrix of the predictors $\mathbf{X}$

Table 5: SCAD for linear regression

Σ_X	n	β^*	FP rate	FN rate	coverage of zero for $\beta = 0$ (adapt[1])	coverage of zero for $\beta = 0$ (oracle[2])	coverage of zero for $\beta^* \neq 0$ (adapt)	coverage of zero for $\beta^* \neq 0$ (oracle)	coverage of 95% CIs of β^* for $\hat{\beta} \neq 0$ (adapt)	coverage of 95% CIs of β^* for $\hat{\beta} \neq 0$ (oracle)
indep	100	0.25	0.183	0.309	0.995	0.943	0.809	0.479	0.636	0.645
indep	200	0.25	0.243	0.020	0.995	0.943	0.423	0.092	0.775	0.927
indep	500	0.25	0.529	0.000	0.988	0.945	0.007	0.000	0.890	0.944
indep	100	0.5	0.276	0.003	0.993	0.927	0.094	0.016	0.824	0.876
indep	200	0.5	0.209	0.000	0.997	0.945	0.000	0.000	0.861	0.918
indep	500	0.5	0.153	0.000	0.995	0.953	0.000	0.000	0.835	0.941
indep	100	1	0.147	0.000	0.996	0.946	0.000	0.000	0.898	0.900
indep	200	1	0.149	0.000	0.999	0.952	0.000	0.000	0.933	0.933
indep	500	1	0.054	0.000	0.999	0.956	0.000	0.000	0.948	0.946
block	100	0.25	0.100	0.144	0.996	0.961	0.714	0.519	0.817	0.802
block	200	0.25	0.157	0.021	0.999	0.960	0.402	0.246	0.885	0.928
block	500	0.25	0.144	0.000	0.995	0.960	0.055	0.011	0.873	0.947
block	100	0.5	0.123	0.013	0.995	0.956	0.225	0.072	0.799	0.900
block	200	0.5	0.171	0.000	0.996	0.958	0.012	0.002	0.857	0.933
block	500	0.5	0.117	0.000	0.997	0.964	0.000	0.000	0.875	0.949
block	100	1	0.163	0.000	0.994	0.945	0.001	0.000	0.880	0.906
block	200	1	0.143	0.000	0.998	0.960	0.000	0.000	0.942	0.933
block	500	1	0.045	0.000	1.000	0.971	0.000	0.000	0.947	0.945
AR	100	0.25	0.129	0.233	0.995	0.955	0.770	0.528	0.734	0.695
AR	200	0.25	0.177	0.051	0.997	0.958	0.476	0.248	0.824	0.895
AR	500	0.25	0.334	0.007	0.992	0.947	0.081	0.017	0.842	0.935
AR	100	0.5	0.168	0.032	0.993	0.948	0.259	0.085	0.760	0.864
AR	200	0.5	0.190	0.000	0.996	0.953	0.018	0.001	0.833	0.920
AR	500	0.5	0.177	0.000	0.996	0.959	0.000	0.000	0.859	0.945
AR	100	1	0.167	0.000	0.994	0.946	0.001	0.000	0.879	0.906
AR	200	1	0.152	0.000	0.999	0.958	0.000	0.000	0.937	0.929
AR	500	1	0.049	0.000	1.000	0.967	0.000	0.000	0.954	0.948

FP - false positive; FN - false negative

[1] corresponds to Algorithm 1, and [2] to Algorithm 2 in the text

Σ_X is the covariance matrix of the predictors $\mathbf{X}$

Table 6: Penalized linear regression

Σ_X	n	β^*	FP rate	FN rate	coverage of zero for $\beta = 0$ (adapt[1])	(oracle[2])	$\beta^* \neq 0$ (adapt)	(oracle)	coverage of 95% CIs of β^* for $\hat{\beta} \neq 0$ (adapt)	(oracle)
indep	100	0.25	0.326	0.162	0.999	0.946	0.930	0.467	0.803	0.797
indep	200	0.25	0.422	0.010	0.996	0.939	0.440	0.109	0.837	0.924
indep	500	0.25	0.485	0.000	0.991	0.945	0.005	0.000	0.873	0.942
indep	100	0.5	0.285	0.002	0.998	0.940	0.213	0.017	0.787	0.901
indep	200	0.5	0.351	0.000	0.995	0.939	0.000	0.000	0.824	0.926
indep	500	0.5	0.434	0.000	0.992	0.945	0.000	0.000	0.861	0.941
indep	100	1	0.247	0.000	0.998	0.944	0.000	0.000	0.792	0.902
indep	200	1	0.327	0.000	0.996	0.944	0.000	0.000	0.828	0.929
indep	500	1	0.432	0.000	0.992	0.944	0.000	0.000	0.866	0.941
block	100	0.25	0.253	0.044	0.999	0.960	0.832	0.557	0.950	0.904
block	200	0.25	0.280	0.006	0.999	0.952	0.390	0.260	0.968	0.936
block	500	0.25	0.342	0.000	0.997	0.944	0.016	0.013	0.961	0.947
block	100	0.5	0.179	0.001	0.999	0.961	0.142	0.073	0.956	0.920
block	200	0.5	0.228	0.000	0.998	0.955	0.002	0.001	0.950	0.933
block	500	0.5	0.299	0.000	0.998	0.945	0.000	0.000	0.949	0.947
block	100	1	0.136	0.000	0.999	0.966	0.000	0.000	0.938	0.917
block	200	1	0.196	0.000	0.998	0.956	0.000	0.000	0.936	0.935
block	500	1	0.286	0.000	0.997	0.946	0.000	0.000	0.940	0.941
AR	100	0.25	0.284	0.109	0.999	0.957	0.882	0.570	0.885	0.840
AR	200	0.25	0.353	0.020	0.999	0.947	0.500	0.263	0.929	0.923
AR	500	0.25	0.396	0.000	0.996	0.948	0.037	0.011	0.919	0.943
AR	100	0.5	0.224	0.012	0.999	0.956	0.249	0.079	0.892	0.905
AR	200	0.5	0.291	0.000	0.998	0.950	0.010	0.001	0.900	0.934
AR	500	0.5	0.364	0.000	0.996	0.948	0.000	0.000	0.912	0.948
AR	100	1	0.189	0.000	0.998	0.958	0.001	0.000	0.867	0.912
AR	200	1	0.261	0.000	0.997	0.950	0.000	0.000	0.886	0.928
AR	500	1	0.341	0.000	0.996	0.947	0.000	0.000	0.904	0.940

FP - false positive; FN - false negative

[1] corresponds to Algorithm 1, and [2] to Algorithm 2 in the text

Σ_X is the covariance matrix of the predictors $\mathbf{X}$

Table 7: Penalized partial least squares linear regression

Σ_X	n	β^*	FP rate	FN rate	coverage of zero for $\beta = 0$ (adapt[1])	coverage of zero for $\beta = 0$ (oracle[2])	coverage of zero for β^* (adapt)	coverage of zero for β^* (oracle)	coverage of 95% CIs of β^* for $\hat{\beta} \neq 0$ (adapt)	coverage of 95% CIs of β^* for $\hat{\beta} \neq 0$ (oracle)
indep	100	0.25	0.001	0.876	0.999	0.999	0.887	0.887	0.111	0.111
indep	200	0.25	0.000	0.861	1.000	1.000	0.862	0.862	0.127	0.127
indep	500	0.25	0.005	0.812	0.999	0.999	0.812	0.812	0.174	0.174
indep	100	0.5	0.000	0.748	1.000	1.000	0.752	0.752	0.232	0.232
indep	200	0.5	0.002	0.707	1.000	1.000	0.707	0.707	0.276	0.276
indep	500	0.5	0.062	0.544	0.995	0.995	0.544	0.544	0.433	0.433
indep	100	1	0.004	0.463	0.998	0.998	0.464	0.464	0.507	0.507
indep	200	1	0.017	0.372	0.997	0.997	0.372	0.372	0.594	0.594
indep	500	1	0.153	0.306	0.990	0.990	0.306	0.306	0.657	0.657
block	100	0.25	0.001	0.784	0.999	0.999	0.784	0.784	0.022	0.022
block	200	0.25	0.000	0.791	1.000	1.000	0.791	0.791	0.006	0.006
block	500	0.25	0.000	0.804	1.000	1.000	0.804	0.804	0.000	0.000
block	100	0.5	0.000	0.713	1.000	1.000	0.713	0.713	0.037	0.037
block	200	0.5	0.000	0.759	1.000	1.000	0.759	0.759	0.002	0.002
block	500	0.5	0.000	0.794	1.000	1.000	0.794	0.794	0.000	0.000
block	100	1	0.003	0.589	0.999	0.999	0.589	0.589	0.088	0.088
block	200	1	0.001	0.651	1.000	1.000	0.651	0.651	0.021	0.021
block	500	1	0.001	0.693	1.000	1.000	0.693	0.693	0.013	0.013
AR	100	0.25	0.002	0.789	0.999	0.999	0.790	0.790	0.051	0.051
AR	200	0.25	0.001	0.773	1.000	1.000	0.773	0.773	0.030	0.030
AR	500	0.25	0.001	0.763	1.000	1.000	0.763	0.763	0.019	0.019
AR	100	0.5	0.003	0.665	0.998	0.998	0.666	0.666	0.117	0.117
AR	200	0.5	0.005	0.649	0.998	0.998	0.649	0.649	0.099	0.099
AR	500	0.5	0.024	0.443	0.992	0.992	0.443	0.443	0.305	0.305
AR	100	1	0.022	0.422	0.990	0.990	0.422	0.422	0.333	0.333
AR	200	1	0.041	0.288	0.986	0.986	0.288	0.288	0.473	0.473
AR	500	1	0.076	0.153	0.982	0.982	0.153	0.153	0.642	0.642

FP - false positive; FN - false negative

[1] corresponds to Algorithm 1, and [2] to Algorithm 2 in the text

Σ_X is the covariance matrix of the predictors $\mathbf{X}$

Table 8: Performance of the various methods when the error distribution in the linear model was a t-distribution with 15 degrees of freedom for Σ_X set ot the identity matrix

Algorithm	n	β^*	FP rate	FN rate	coverage of zero for $\beta = 0$ (adapt[1])	(oracle[2])	$\beta^* \neq 0$ (adapt)	(oracle)	coverage of 95% CIs of β^* for $\hat{\beta} \neq 0$ (adapt)	(oracle)
lars	100	0.25	0.19	0.31	1.00	0.95	0.88	0.52	0.65	0.65
lars	100	0.5	0.29	0.00	1.00	0.93	0.20	0.03	0.80	0.89
lars	200	0.25	0.30	0.03	1.00	0.94	0.47	0.14	0.82	0.92
lars	200	0.5	0.32	0.00	1.00	0.94	0.00	0.00	0.83	0.93
lars	500	0.25	0.34	0.00	1.00	0.95	0.02	0.00	0.83	0.94
lars	500	0.5	0.32	0.00	1.00	0.94	0.00	0.00	0.82	0.94
LASSO	100	0.25	0.19	0.31	1.00	0.94	0.87	0.52	0.65	0.65
LASSO	100	0.5	0.29	0.00	1.00	0.93	0.20	0.03	0.80	0.89
LASSO	200	0.25	0.31	0.03	1.00	0.94	0.47	0.14	0.83	0.92
LASSO	200	0.5	0.32	0.00	1.00	0.94	0.00	0.00	0.83	0.93
LASSO	500	0.25	0.33	0.00	1.00	0.95	0.02	0.00	0.83	0.94
LASSO	500	0.5	0.33	0.00	1.00	0.94	0.00	0.00	0.83	0.94
elastic net	100	0.25	0.28	0.27	1.00	0.95	0.87	0.56	0.71	0.70
elastic net	100	0.5	0.45	0.01	0.99	0.95	0.28	0.05	0.82	0.92
elastic net	200	0.25	0.37	0.03	0.99	0.95	0.47	0.15	0.83	0.93
elastic net	200	0.5	0.43	0.00	0.99	0.95	0.01	0.00	0.76	0.94
elastic net	500	0.25	0.37	0.00	0.99	0.95	0.02	0.00	0.82	0.95
elastic net	500	0.5	0.37	0.00	0.99	0.95	0.00	0.00	0.72	0.94
relaxo	100	0.25	0.15	0.40	0.99	0.95	0.76	0.56	0.58	0.56
relaxo	100	0.5	0.19	0.02	0.98	0.95	0.07	0.04	0.87	0.90
relaxo	200	0.25	0.18	0.09	0.99	0.95	0.32	0.16	0.85	0.87
relaxo	200	0.5	0.05	0.00	0.98	0.98	0.00	0.00	0.94	0.94
relaxo	500	0.25	0.05	0.01	0.98	0.98	0.01	0.01	0.94	0.95
relaxo	500	0.5	0.01	0.00	0.99	0.99	0.00	0.00	0.95	0.95
penalized.pls	100	0.25	0.00	0.88	1.00	1.00	0.89	0.89	0.10	0.10
penalized.pls	100	0.5	0.00	0.76	1.00	1.00	0.77	0.77	0.22	0.22
penalized.pls	200	0.25	0.00	0.87	1.00	1.00	0.87	0.87	0.11	0.11
penalized.pls	200	0.5	0.00	0.72	1.00	1.00	0.72	0.72	0.26	0.26
penalized.pls	500	0.25	0.01	0.82	1.00	1.00	0.82	0.82	0.16	0.16
penalized.pls	500	0.5	0.04	0.58	1.00	1.00	0.58	0.58	0.39	0.39
SCAD	100	0.25	0.82	0.04	1.00	0.94	0.88	0.59	0.94	0.90
SCAD	100	0.5	0.38	0.00	0.99	0.93	0.14	0.04	0.86	0.89
SCAD	200	0.25	0.96	0.00	0.96	0.95	0.28	0.19	0.96	0.95
SCAD	200	0.5	0.31	0.00	0.99	0.94	0.00	0.00	0.87	0.93
SCAD	500	0.25	1.00	0.00	0.92	0.95	0.01	0.00	0.92	0.95
SCAD	500	0.5	0.15	0.00	0.99	0.95	0.00	0.00	0.83	0.94

FP - false positive; FN - false negative

[1] corresponds to Algorithm 1, and [2] to Algorithm 2 in the text
